# Human iPSC-Derived Neurons as A Platform for Deciphering the Mechanisms behind Brain Aging

**DOI:** 10.3390/biomedicines9111635

**Published:** 2021-11-07

**Authors:** Chuan-Chuan Chao, Po-Wen Shen, Tsai-Yu Tzeng, Hsing-Jien Kung, Ting-Fen Tsai, Yu-Hui Wong

**Affiliations:** 1 Aging and Health Research Center, National Yang Ming Chiao Tung University, Taipei 112, Taiwan; chao2200@gmail.com (C.-C.C.); tftsai@ym.edu.tw (T.-F.T.); 2 Department of Neurology, School of Medicine, National Yang Ming Chiao Tung University, Taipei 112, Taiwan; 3 Program in Molecular Medicine, National Yang Ming Chiao Tung University and Academia Sinica, Taipei 112, Taiwan; janothan1983@gmail.com; 4 Ph.D. Program for Cancer Biology and Drug Discovery, College of Medical Science and Technology, Taipei Medical University, Taipei 110, Taiwan; 5 Cancer Progression Research Center, National Yang Ming Chiao Tung University, Taipei 112, Taiwan; tytzeng@nycu.edu.tw; 6 Institute of Molecular and Genomic Medicine, National Health Research Institutes, Zhunan, Miaoli 350, Taiwan; hsingjienkung@gmail.com; 7 Research Center of Cancer Translational Medicine, Taipei Medical University, Taipei 110, Taiwan; 8 Department of Biochemistry and Molecular Medicine, Comprehensive Cancer Center, University of California at Davis, Sacramento, CA 95817, USA; 9 Department of Life Sciences and Institute of Genome Sciences, National Yang Ming Chiao Tung University, Taipei 112, Taiwan; 10 Brain Research Center, National Yang Ming Chiao Tung University, Taipei 112, Taiwan

**Keywords:** brain aging, neuronal senescence, human induced pluripotent stem cells (hiPSCs), induced neurons (iNs), CRISPR, genome editing technology

## Abstract

With an increased life expectancy among humans, aging has recently emerged as a major focus in biomedical research. The lack of in vitro aging models—especially for neurological disorders, where access to human brain tissues is limited—has hampered the progress in studies on human brain aging and various age-associated neurodegenerative diseases at the cellular and molecular level. In this review, we provide an overview of age-related changes in the transcriptome, in signaling pathways, and in relation to epigenetic factors that occur in senescent neurons. Moreover, we explore the current cell models used to study neuronal aging in vitro, including immortalized cell lines, primary neuronal culture, neurons directly converted from fibroblasts (Fib-iNs), and iPSC-derived neurons (iPSC-iNs); we also discuss the advantages and limitations of these models. In addition, the key phenotypes associated with cellular senescence that have been observed by these models are compared. Finally, we focus on the potential of combining human iPSC-iNs with genome editing technology in order to further our understanding of brain aging and neurodegenerative diseases, and discuss the future directions and challenges in the field.

## 1. Introduction

People worldwide are living longer because of improvements in medicine and public health. The World Health Organization estimates that the world population over 60 years old will nearly double from 12% to 22% between 2015 and 2050, reaching nearly 2.1 billion individuals (https://www.who.int/ accessed on 1 May 2017). As the elderly population increases, the financial burden of age-related health disorders will also increase, and effective preventive and/or therapeutic approaches are urgently needed. Among various age-related diseases, neurodegeneration, and the associated cognitive decline, is particularly relevant owing to its great influence on health span and quality of life [1].

As age is inherently linked to an increased predisposition to many diseases as well as an increased likelihood of death, people have been searching for ways to delay aging for centuries. According to historical records, Qín Shǐ Huáng, the first emperor who unified ancient China and who laid the foundation of the Great Wall of China, launched an obsessive search for the elixir of life before dying at the age of 50 in 210 B.C.E. Elsewhere at about the same time, Alexander the Great, who conquered most of the known western world before he died aged 32 around 323 B.C., may have been looking for the legendary Fountain of Youth that healed the ravages of war wounds and age. Unfortunately, such legendary artifacts have remained just legendary up to the present, and reversing the aging process resides, as yet, in the territory of fairy tales. However, the search for the means to delay aging has not ceased and it is now being performed by biologists rather than servants of rulers of vast kingdoms. These researchers have been working to understand the mechanisms that underlie the aging process so that we may be able to delay aging or even potentially reverse it, at least to some degree [2,3,4,5].

The prolonged lifespan presents unique challenges to many of the postmitotic cells that make up our bodies, and this results in us being highly susceptible to age-associated disorders, including various neurodegenerative diseases. Animal studies, both in vivo and in vitro, have provided significant insights into the molecular mechanisms associated with senescent neurons and brain aging [6,7]. However, animal models of brain disorders have been found to not necessarily reflect the complexity of various human conditions, and unfortunately have not been very predictive when evaluating drug candidates for several diseases. For example, Alzheimer’s disease (AD) is the most common type of dementia and also one of the leading causes of death worldwide [8]. Nonetheless, decades of animal research have failed to translate into any noteworthy advances that help to prevent or treat AD [9]. In view of this failure, a different and human-relevant approach is critically needed. In this review, we will discuss what constitutes neuronal senescence and the evidence implicating senescence in brain dysfunction; we will also explore the current in vitro cell models that are available for the study of neuronal aging. The latter promises to be a useful strategy for the development of novel therapeutics that treat the pathologies associated with brain aging.

## 2. The Signaling Pathways Associated with Neuronal Senescence and Brain Aging

**Figure 1:**
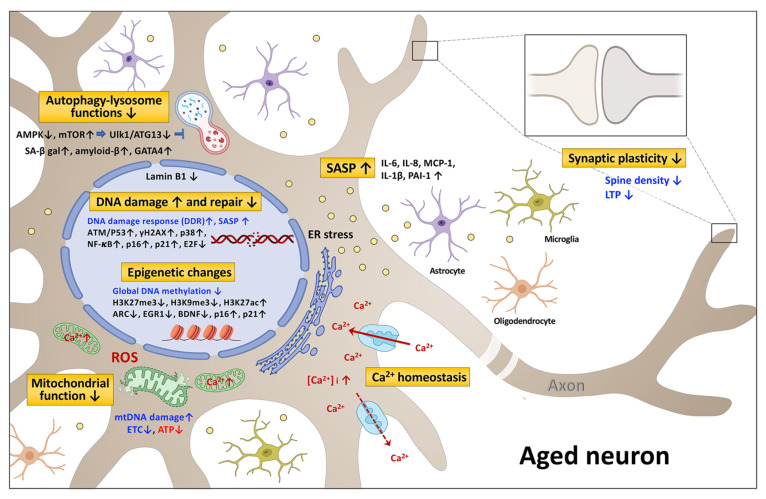
A schematic diagram of the signaling pathways associated with neuronal senescence and brain aging. Altered expression or activity of notable genes (black) in aged neurons are shown by up or down arrows, which indicate up or down regulation, respectively. The cellular processes affected are marked in blue. The metabolites are marked in red. The seven resulting brain-related functional changes are depicted. ER, endoplasmic reticulum; ROS, reactive oxygen species; SASP, senescence associated secretory phenotype. The figure was created with Biorender.com accessed on 19 October 2021.

Cells are the basic building blocks of all multicellular organisms. Cellular senescence is thought to contribute to brain aging and age-related neurodegenerative diseases through a variety of mechanisms [10,11]. Nevertheless, there are common signaling pathways and hallmarks [12,13,14]; these include the DNA damage response, chromatin alterations, mitochondria impairment, lysosome impairment, autophagy disruption, SASP (senescence associated secretory phenotype) changes, and decreased synaptic plasticity—all of which can be recognized in senescent neurons (Figure 1). Each of these domains is activated during aging, and all appear to interact with each other. Cell senescence has been identified as an important driver of mammalian brain aging [15,16,17].

### 2.1. DNA Damage and Repair

The genomic integrity of an individual is constantly challenged by DNA replication errors, spontaneous hydrolysis reactions, and reactive oxygen species (ROS), all of which lead to nucleotide changes, single-strand breaks (SSB), and double-strand breaks (DSB). The DNA damage response (DDR) promotes DNA repair and is a guardian of our genome [18,19,20]. The DNA repair processes, which require a series of catalytic reactions mediated by multiple proteins, are affected by age [18]. It was reported that the activity level of non-homologous end joining (NHEJ) gradually decreases with age [21]. When key proteins in the DDR are defective, accelerated aging ensues; this is due to an accumulation of mutations that eventually cause cellular malfunctions, senescence, and apoptosis [22]. Indeed, unrepaired DNA damage and DNA mutations accumulate in an age-related manner [23,24]. Cells carrying mutations at defined loci have also been shown to increase with age in humans and mice [24,25]. The ATM mutation, which is involved in DSB signaling/repair [26]; the ERCC1 and XPF mutations, which are involved in nucleotide excision repair (NER) and crosslink repair [27]; and the XPA/CSB mutation, which is also involved in NER, have been identified in the brain tissue of prematurely aging mice [28]. Recently, single-cell whole-genome sequencing has been used to perform genome-wide somatic single-nucleotide variant (sSNV) identification across the human genome [29]. The presence of C > A variants has been shown to modestly increase in neurons with age and is known to be most closely associated with oxidative DNA damage [30]. These C > A variants are also known to be present in the neurons of individuals with Cockayne syndrome (CS) and xeroderma pigmentosum (XPC). sSNVs increase almost linearly with age in neurons obtained from the prefrontal cortex and the hippocampus of CS and XPC individuals; they are also more abundant in individuals with neurodegenerative diseases. 

DDR genes comprise less than 10% of the genes significantly affected by age in mice [31]. The expression of ATM (ataxia telangiectasia mutated), a DDR gene and a key initiator of DNA repair, is decreased in old mouse fibroblasts and brain tissues [32,33]. In mice with ataxia telangiectasia, the ATM mutation results in DNA repair deficiency and an accumulation of DNA fragments; this activates the cyclic GMP–AMP synthase (cGAS)-stimulator of interferon genes (STING) signaling, a DNA fragment sensor system, and triggers chronic inflammation as well as premature senescence in the central nervous system (CNS) [34]. Telomere repeat binding factor 2 (TRF2), which is essential for maintaining the structure and function of telomeres, is down-modulated and impaired during aging. This dysfunctionality causes DNA damage, ATM activation, γH2AX accumulation, p21 induction, and increased β-galactosidase activity in primary mouse embryonic hippocampal neurons and in human SH-SY5Y cells [35,36,37]. Severe DNA damage activates a whole host of responses in aged mice that affect the cortex, hippocampus, peripheral neurons, and Purkinje neurons, including p38 MAP kinase activation, ROS production oxidative damage, heterochromatinization, IL-6 production, p21 induction, and increased β-galactosidase activity (Figure 1). Furthermore, senescent-like neurons increase with age [38]. Similarly, the altered expression of genes involved in the cell cycle (p53, CDKN1A, CCND1), in inflammation (IL1B, IL6, PTGS2, SERPINE1), in stress response (p38 MAP kinase), and in DDR (CHEK1) are present in the hippocampus of AD patients as well as in that of old mice and old rats [39]. Higher levels of p53 and p21 have been found in the brains of RAD6B-deficient mice that show signs of neuronal aging and degeneration [40]. RAD6B participates in DNA DSB repair through the ubiquitination of histone H2B, and a deficiency in this protein leads to genome instability, which is associated with increased neurodegeneration and increased memory loss. The information summarized above suggests that DNA repair genes are critical to various DNA damage responses, and deficiencies in these genes bring about neuronal aging and its related disorders.

### 2.2. Epigenetic Changes

Gene expression in the aging brain depends on the interaction of the brain’s genetic and epigenetic programing. Epigenetic modifications, which include alterations of DNA methylation as well as histone modifications, result in a remodeling of chromatin structure and a reprograming of gene expression [41]. These processes have been shown to be related to aging, neuropathology, and the progression of various neurodegenerative diseases [42,43,44].

#### 2.2.1. DNA Methylation

DNA methylation, one of the most important epigenetic modifications, is closely involved in tissue-specific gene expression and the silencing of transposable elements. The DNA methyltransferase family (DNMT1, DNMT3a, and DNMT3b) adds a methyl group to the 5-carbon of cytosine to form 5-methylcytosine (5mC), often next to a guanine nucleotide (a CpG site). CpG methylation has been shown to be related to neuroplasticity in neuronal cells [45,46]. However, in various studies of brain aging, it has been pointed out that the methylation of non-CpG sites, such as CpA, CpT, and CpC, seem to be involved in cognition deficits [47,48]. DNA methylation primarily inhibits gene expression. The reverse process, DNA demethylation, begins with the formation of 5-hydroxymethylcytosine (5hmC) by ten-eleven translocation (TET) enzymes. The 5hmC phenomenon is generally associated with increased gene expression. In the mouse and human brain, this form of cytosine is enriched in synaptic genes and has a higher level in adult neurons [48,49]. The effects of 5hmC on neuronal senescence and the aging of the brain remains to be established [50].

Several lines of evidence suggest that DNA methylation plays a role in the expression of genes involved in learning and the memory of adult CNS, and alterations to the expression levels of these genes seem to bring about memory loss and cognitive impairment during the brain aging process [51,52,53,54,55,56,57,58,59]. In previous studies, the global hypomethylation of the genome were detected with age [60,61]. Hypomethylation of the promoters of *p16^INK4A^* and *p21^CIP1/WAF1^*, two cell-cycle inhibitors and senescence markers, may play a role in cellular senescence and aging. In naturally aging mouse models or human tissues, the increased level of p21 and p16 proteins in the old group as compared to the young group was observed [62,63]. It was experimentally determined that the cause of such an increase was due to the reduced methylation of the promoter regions of the genes. There are 5 and 10 CpG islands found within 50 kbp upstream of the *p16^INK4A^* and *p21^CIP1/WAF1^* promoter regions of human cells, respectively, and DNA methylation mostly occurred in these CpG island regions, which dictate the gene expression. The inhibition of DNMT-mediated DNA methylation by 5-azacytidine (5-AzaC) or siRNA induced cellular senescence and cell-cycle arrest in human primary cells, which was accompanied by a reduction of the methylation level of the CpG islands upstream of the promoter region of *p16^INK4A^* and *p21^CIP1/WAF1^*, and by the upregulation of p16 and p21 expression. This phenomenon was also observed in prolonged cultured cells as compared to early-passage cells. These studies suggest that hypomethylation may be an underlying contributor to cellular senescence and aging [64,65]. On the other hand, a positive correlation exists between aging and hypermethylation of specific promoters. For instance, *Arc* (activity-regulating cytoplasmic genes) and *Egr1* promoters in the neurons of old rats were found to have a higher degree of methylation, and in agreement with this, the expression levels of the corresponding genes were decreased; this seemed to result in memory decline [58,59]. Similarly, the methylation of the REST promoter and the resulting suppression of expression set up a cascade of epigenetic remodeling [66] that led to an impairment of the cognitive functioning of the aging brain and the development of neurodegenerative diseases [67,68]. Likewise, the level of KLOTHO—a glycosylated transmembrane protein that is expressed in the choroid plexus and neurons of the brain as well as an anti-aging factor—decreased with age [69]. This was, in part, due to hypermethylation of 36 residues at six CpGs in the promoter of old animals [70,71,72]. In addition, neurodegenerative diseases such as AD and Parkinson’s disease (PD) have common abnormal DNA methylation profiles, which largely seem to affect the expression of key genes involved in various pathological pathways, including those associated with amyloid plaques and neurofibrillary tangles (NFTs) [43,73,74].

#### 2.2.2. Histone Modification

The post-translational modification of histones is also an epigenetic mechanism that controls the expression of genes related to brain functioning [43,75]. Via the post-translational modification of the N-terminal tail of histones, the structure of chromatin can be changed; this allows enzymes that promote or inhibit transcription to be recruited, which further modifies the chromatin. There are many types of histone modification. Among them, methylation and acetylation seem to be the ones most related to aging and age-related diseases [76,77,78,79,80,81]. In various species, the methylation or acetylation status of specific lysine residues on H3 and H4 has been found to have an important influence on aging and diseases related to aging [82].

Previous studies showed that the primary changes in gene expression during aging occur via a reduction in inhibitory markers (H3K9me3 and H3K27me3) and an accumulation of activating markers (H3K27ac) [80,83,84]. Decreased levels of lamin B1, H3K27me3, and H3K9me3 lead to a decrease in heterochromatin content, which has been found to be related to aging [85]. The recruitment of EZH2, a key methylase of the H3K27 residue in the promoter regions of *p16^INK4A^* and *p21^CIP1/WAF1^*, are reduced in senescent cells, which could account for the decrease of suppressive histone marks in the promoter regions and the increase of p16 and p21 expression in senescent cells [64]. While there seems to be an overall reduction in repressive histone markers such as H3K9me3, a regional increase in H3K9me3 on specific promoters, such as the *brain-derived neurotrophic factor (bdnf)*, has been detected during aging; the latter results in the diminished expression of BDNF [86,87]. In addition to the levels of H3K27me3 and H3K9me3 changing with age, there are other histone modifications altered during cell senescence. For example, H3K9me, H3K9ac, H4K20me, H4K20me2, H3.3, and H2A.2 are increased, while H3K9me2, H3.1, H3.2, H4, and H2A.1 are decreased during the aging process [88]. It is currently unclear how physiological aging changes the landscape of inhibitory histone methylation. Nonetheless, changes in overall histone methylation does not seem to determine the aging process, while the site-specific methylation of aging-related genes also seems to be important. Further research is needed to fully understand how changes in the histones present in heterochromatin are related to brain aging.

The decrease in repressive histone methylation by H3K27 paves the way for the acetylation of the histone. H3K27ac is a marker that decorates the enhancers and promoters of active genes. The acetylation and deacetylation of histones are carried out by histone acetyltransferase HAT and deacetylases HDAC, respectively. It has been reported that the expression of these related regulatory molecules changes in the elderly brain, and thus may be associated with age-related changes in gene transcription [76]. Likewise, lysine acetyltransferase (KAT) and lysine deacetylase (KDAC) are also involved in acetylation/deacetylation. Among them, Sirtuin 1 (SIRT1), a KDAC, is well recognized as a regulatory protein related to aging. SIRT1 is regulated by many biological processes, including damage repair [89]. The expression level and activity of SIRT1 decline during aging; this increases NF-κB transcription factor activity, which is required for the transactivation of SASP members [90]. In addition, it has been found that the level and activity of HDAC2 is increased in the hippocampus of aged mice, and that HDAC2 recruitment is enriched on neuron-related genes such as Arc, Egr1, Homer1, and Narp. The reduction of the acetylation of H3K9 and H3K14 leads to the reduced expression of these genes, which then affects nerve functionality [91,92]. Other studies have indicated that a decrease in H3K27ac near the *bdnf* promoter may be related to the increase in HDAC2 and HDAC4 in old hippocampal neurons [93]. In summary, DNA methylation and histone modifications are intertwined, and seem to bring about some of the changes in gene expression observed during brain aging.

### 2.3. Mitochondrial Dysfunction

Mitochondria are the major sites of bioenergy production in eukaryotes, and they are involved in pyruvate oxidation, the TCA cycle, electron transfer, adenosine triphosphate (ATP) production, cytoplasmic calcium buffering, and various ROS-mediated signaling pathways [94,95,96,97]. Mitochondrial function becomes impaired as they age, and this leads to changes in mitochondrial respiration and to a reduction in energy (ATP) production, as well as to extensive changes in the levels of metabolites related to mitochondrial function [98,99]. It has long been held that aging and aging-related degenerative diseases are the result of free radicals, which interconvert with ROS and attack cells and tissues [100,101]. ROS is also formed physiologically during OXPHOS (oxidative phosphorylation) and the energy production process of mitochondria [99]. Toxic ROS, when produced during old age due to a homeostatic imbalance related to excessive cellular oxidative stress, leads to damage in the mtDNA and in the mitochondria themselves; this can be followed by a mitochondrial crisis. Such a crisis can involve respiratory chain deficiency, ATP synthesis disorder, an increase in calcium concentrations, lipid peroxidation, protein denaturation, and protein aggregation [102,103]. These events ultimately result in cell apoptosis and brain aging [82]. A significant increase in ROS production has been observed in aged neurons, and there also seems to be an increase in the markers associated with neuronal senescence, including higher expression levels of p53 and the increased activation of phosphor-JNK and phosphor-p38 MAPK [100,104]. A decrease in neurite outgrowth in older neurons has been detected in primary cortical neurons isolated and cultured from adult mice [105]. Aged neurons exhibit respiratory dysfunction, decreased mitochondrial membrane potential, changes in mitochondrial membrane transport proteins, and mitochondrial calcium accumulation [106]. These findings suggest that the presence of dysfunctional mitochondria in aged neurons may be related to an age-dependent reduction in neuronal functions. In support of this hypothesis, an mtDNA 3860-bp deletion was found to be common in the auditory nervous system of mice, and its presence increases with age; the presence of this deletion may contribute to age-related hearing loss [107].

The decline in mitochondrial function caused by an aging brain is related to a decrease in the level of NAD+ and changes in the ratio of NAD+/NADH in the cell; this affects the activity of NAD+ dependent enzymes, which are essential for the functioning of neurons. These enzymes include SIRT1, an NAD-dependent deacetylase [108,109,110]. SIRT1 and AMPK, which activate PGC-1α/β, are the coactivators of the peroxisome proliferator-activated receptor γ (PPARγ); they play an essential role in the biogenesis of mitochondria [111]. In this context, PGC-1α has been suggested to exert neuroprotective effects against 1-methyl-4-phenyl-1,2,3,6- tetrahydropyridine (MPTP)-induced PD [112,113]. Increasing the levels of PGC-1α, FNDC5, or BDNF in Neuro-2a cells is able to counteract the effect of Aβ1-42 oligomers during neuronal apoptosis, and this effect is believed to occur via an improvement in mitochondrial functioning [114,115]. PGC-1α modulates mitochondrial function via the activation of the expression of ERRα, NRF-1, and PPARγ, which are the transcription factors responsible for the expression of various nuclear genes involved in mitochondrial biogenesis. These findings provide additional evidence for a link between mitochondrial dysfunction and neuronal aging.

The occurrence of sarcopenia and muscle atrophy in the elderly may be related to the loss of motor neurons [116]. The loss of spinal cord motor neurons with age is characterized by a deficiency in mitochondrial respiratory complex I, decreased mtDNA, and a smaller soma size, which suggests that mitochondrial dysfunction in aged motor neurons leads to cell loss and the denervation of the skeletal muscle. An impairment of mitochondrial quality is known to change mitochondrial transport in axons [117,118]. Newly generated mitochondria are transported anteriorly from the soma to fill the axon mitochondrial pool, while damaged mitochondria are transported retrogradely for repair or degradation. Abnormal mitochondrial transport can thus lead to mitochondrial dysfunction and axon degeneration in various neurological and psychiatric diseases [119,120,121]. Using neurons derived from human iPSCs obtained from PD patients that were carrying the *SNCA* gene duplication, it was found that the formation of oligomeric α-Syn leads to a decrease in axonal mitochondrial transport [122]. α-Syn oligomerization impairs anterograde axonal transport via the subcellular relocation of the transport regulators Miro1, KLC1, and Tau, as well as through a decrease in ATP levels. These findings indicate that there is a connection between mitochondrial dysfunction and aging-related neuronal diseases.

### 2.4. Autophagy-Lysosome Dysfunction

Autophagy is a lysosomal-mediated degradative process that affects nucleic acids, proteins, lipids, and organelles; it plays a critical role in cellular and tissue homeostasis [123,124], including differentiation, development, and aging [125,126]. By degrading non-functional proteins and retired organelles, autophagy maintains a healthy supply of metabolic energy. The crucial role that energy metabolic rate plays in the aging process was acknowledged over a century ago, and it is well known that metabolism tends to slow down with age, resulting in an energy imbalance [127,128]. Thus, the regulation of autophagy and lysosomal functioning is expected to be linked to aging processes. Indeed, autophagic activity declines in *C. elegans*, *Drosophila*, mouse, and human cells as they age, and this is accompanied by a decrease in the expression of various autophagy-related genes, such as *ATG5*, *ATG7*, and *BECN1* in humans and *Atg2*, *Atg8a*, and *Bchs* in mice and *Drosophilla* [129,130,131,132]. In aged wild-type mice, autophagy is diminished in neuronal cells, as evidenced by a decrease of autophagolysosomal fusion, and the fact that there is an impaired delivery of autophagy substrates to lysosomes in the hypothalamus [133]. Decreased lysosomal activity has also been shown to be associated with the aging process and age-associated diseases [134]. The accumulation of β-galactosidase and GATA4 in senescent cells is in part due to the impaired degradation of these molecules (see below) [135,136]. Functional studies using laboratory animals and humans support the essential role of autophagy in neural protection, anti-inflammation activity, genomic integrity, and cellular/tissue homeostasis [126,137]. In addition, age-dependent autophagy deficits have been found to be closely associated with several neurodegenerative diseases [138,139]. These studies collectively suggest that autophagy and lysosome impairment contribute to brain aging.

Knowledge on the cellular pathways that modulate autophagy is likely to provide insights into the brain aging process and also help to identify the modulators of the aging processes. Nutrient deprivation is well recognized to be capable of inducing autophagy, and dietary or caloric restriction has been widely shown to extend the lifespan of rodents and to delay the aging process in other organisms [140,141,142,143,144,145,146,147]. The principal pathways by which caloric restriction mediates life-extension are the nutrient sensors AMPK and mTOR [148,149,150,151]. These pathways negatively impact each other, with AMPK being an inducer of autophagy and mTOR being an inhibitor of autophagy. AMPK is activated by AMP; it is an energy-sensing serine/threonine kinase that responds to a decrease in cellular energy levels and is activated by caloric restriction [152,153,154]. mTOR, which is also a serine/threonine protein kinase, forms two multiple protein complexes in mammalian cells, mTORC1 and mTORC2 [155,156]. mTOR is suppressed by nutrient starvation and inhibits autophagy and lysosomal functions, thus serving as a regulator of longevity and aging in mice [157,158,159,160]. AMPK directly phosphorylates the autophagy initiator ULK1 (Unc-51-like kinase 1), and this induces autophagy [161,162]. AMPK is also able to regulate autophagy indirectly via the inhibition of mTORC1 through the phosphorylation of the tuberous sclerosis complex 2 (TSC2), and/or through phosphorylation of the regulatory subunit raptor [163,164,165,166]. The activation of AMPK positively promotes autophagy, maintains mitochondrial quality control, reduces insulin resistance, and relieves oxidative stress in AD [167]. Conversely, mTOR directly phosphorylates ULK1 and ATG13, which inhibits the activity of the autophagosome initiation complex [168]. Metformin, which is known to activate AMPK by impairing mitochondrial ATP production, has been shown to cause an anti-aging effect via autophagy induction [168,169]. On the other hand, rapamycin, an mTOR inhibitor that induces autophagy, has also been shown to extend the lifespan of wild-type mice [170,171,172]. Rapamycin also protects against neuronal death when studied using in vitro and in vivo models of neurodegenerative diseases [173,174]. When taken together, the abovementioned studies collectively demonstrate the importance of autophagy and lysosomes during the brain aging process, and the potential beneficial impact of modulators that are able to activate AMPK and suppress mTOR, both of which induce autophagy during an organism’s lifespan.

### 2.5. Senescence-Associated Secretory Phenotype (SASP)

In addition to the findings above, another important feature of cellular senescence involves the complex secretion of inflammatory cytokines, chemokines, growth factors, ROS, and metalloproteinases; these are collectively known as SASP [175]. Previous studies have provided evidence that the components of the SASP are able to modulate many of the physiological consequences that affect senescent cells, and do so in both a paracrine and an autocrine manner [176]. Regarding senescent brain cells, the expression of various SASP factors, such as IL-6 and/or monocyte chemotactic protein 1 (MCP-1), has been found to be significantly higher in long-term cultured cortical neurons than in cells cultured for only a few days [177,178]. Notably, this IL-6 induction is correlated with the up-regulation of the REST protein, which is a marker of neuronal senescence. In brain tissue, IL-6 production, along with an increase in the activity of the senescence-associated enzyme beta-galactosidase (SA-β-gal), has been observed in the cortical and Purkinje neurons of old mice [38]. The GATA4 transcription factor has been identified as a key activator of SASP genes [135]. GATA4 accumulates during cellular senescence, mainly due to its increased protein stability. Interestingly, GATA4 stability has been found to be regulated by autophagy. The autophagic receptor protein p62/SQSTM1 mediates the degradation of GATA4 under normal conditions. However, once the cell experiences senescence-inducing stimuli, the interaction between GATA4 and p62 decreases, and GATA4 escapes autophagic degradation and begins to accumulate. This accumulation of GATA4 initiates a transcriptional circuit that activates NF-κB and the SASP. Furthermore, the molecules that form the SASP have both an autocrine role, fostering the senescent phenotype, and a paracrine role, inducing senescence in the surrounding cells. For example, it has been shown that a conditioned medium from senescent cortical cells can induce paracrine senescence in co-cultured mouse embryonic fibroblasts (MEFs) [178]. Taken together, these findings suggest that senescent neuronal cells not only develop a functional SASP phenotype, but they can also spread the senescence phenotype via various secreted factors to surrounding cells such as astrocytes, microglia, or endothelial cells.

### 2.6. Calcium Homeostasis and Synaptic Plasticity

Age-associated cognitive decline is an important human experience that differs in extent between individuals. Long-term potentiation (LTP) is the most remarkable form of synaptic plasticity, and this is characterized by a persistent increase in synaptic efficacy following the tetanic stimulation of an afferent pathway to one of the hippocampal sub-regions. On the basis of its properties, LTP has been proposed as a biological substrate for learning and/or memory. Although many of its exact molecular and cellular mechanisms have yet to be fully understood, there is consensus that alterations in neuronal calcium homeostasis contribute to age-related deficits in learning and memory. Indeed, several lines of evidence show that the age-related changes in calcium homeostasis are driven, at least in part, by changes in the expression of calcium channels. One of the first experiments to support this hypothesis found that there was an age-related increase in calcium spike duration, and that this can be used as a measure of calcium influx via the voltage-gated calcium channels (VGCCs) [179]. Later, Thibault and Landfield’s group demonstrated that CA1 pyramidal neurons from aged animals have an enhanced L-type VGCC density when compared to young and middle-aged rats [180]. More importantly, this increase in current density correlated with a decrease in Morris water-maze performance, which suggests that the up-regulation of channel density and calcium spike duration contributes significantly to the age-related decline that affects hippocampus-dependent learning [180]. In addition to these, it has been demonstrated that age-induced autophagy disturbances and various SASP components can also bring about hippocampal LTP impairment, and that this is associated with depressive-like behaviors and cognitive deficits in old animal models [181,182]. 

Information processing in the brain is a highly complex process, and seems to principally rely on the activity of neurons and their interconnection via synapses. Both dendritic morphology and the density of dendritic spines seem to undergo age-associated changes. In the cortical neurons of animals and humans, numerous studies have shown that there is a regression of the dendritic arbors of cortical neurons that comes with age. Total dendritic length and complexity decrease with age when apical and basal dendrites are examined [183]. Moreover, a decrease in dendritic spine density with age has been reported in cortical neurons and in the CA3 hippocampal region, while the spine numbers in the rat CA1 region and the human CA1 region seem to generally remain unchanged [184,185,186]. In summary, aging affects neuronal function and plasticity at different levels, ranging from biochemical changes and biophysical alterations to changes in cell morphology. Despite the fact that these changes are multidirectional and vary across neuronal subtypes and across different brain regions, they all contribute to age-related cognitive deficits.

## 3. Overview of In Vitro Cell Models for Neuronal Senescence

We now explore a series of modeling approaches that illustrate the use of in vitro cell models for studying brain aging (Table 1). Although we cannot comprehensively review all cell-based modeling that target neuronal senescence, these themes are drawn from across the field in order to demonstrate how cell-based models are able to yield new insights into the various scientific problems that are known to have significant cell-scale effects.

### 3.1. Immortalized Cell Lines

Traditional in vitro cell culture techniques for neurological studies have usually involved 2D monolayers grown in standard tissue-culture dishes/plates. These are often based on the use of immortalized cell lines such as murine embryonic carcinoma (P19) cells [215], murine neuroblastoma (Neuro-2a) cells [216], pheochromocytoma (PC-12) cells derived from the rat adrenal medulla [217], or human neuroblastoma SK-N-SH (SH-SY5Y) cells [218]. The P19 cell line, which is pluripotent and can be maintained in an undifferentiated state, can be induced to give derivatives that represent the three germ layers, namely the endoderm, mesoderm, and ectoderm, depending on the treatment used [215]. The treatment of aggregated P19 cells with retinoic acid (RA) results in their differentiation into neurons and glial cells, as identified by antibodies against tubulin, neurofilaments, and GFAP, respectively [219]. The Neuro-2a cell line, which is similar to P19, can also be differentiated into neurons in response to various stimuli, including RA, 12-O-Tetradecanoylphorbol-13-acetate (TPA), dibutyl cyclic AMP (dbcAMP), and a number of trophic factors [220,221,222,223,224]. The differentiated Neuro-2a cells exhibited the characteristics of neurons, including neurite outgrowth and the heightened expression of neuronal markers [225,226]. In contrast to the above, pheochromocytoma PC-12 cells can be induced to differentiate into neuron-like cells by the nerve growth factor (NGF); this involves the expression of neurofilaments and the appearance of neurite outgrowth [227]. More importantly, NGF-treated PC-12 cells release several neurotransmitters, including dopamine, noradrenaline, and acetylcholine [228,229,230]; this means that they can serve as a good cellular model for neuronal differentiation and neurodegeneration [231]. The human SH-SY5Y is the prototype of various cell models that are used to study neuronal differentiation and cellular senescence. Many studies have reported the use of RA to differentiate SH-SY5Y into neuron-like cells. Sequential treatment with RA, BDNF, TPA, and/or dbcAMP has also been used for differentiation into various neuronal subtypes [232,233,234,235]. The morphological changes associated with the various neuronal phenotypes and the expression of a number of neuronal marker genes, such as Tuj1, MAP2, NSE, NeuN, and synaptophysin, are the criteria used when studying such differentiation [236,237,238,239]. Finally, the SH-SY5Y cell line has also been employed to study neurodegenerative diseases, including AD, PD, neurotoxicity, ischemia, and amyotrophic lateral sclerosis (ALS) [207,240,241,242,243,244,245,246,247,248].

The major advantages of the immortalized cell models for neurological research include the following: (1) they are easy to work with and are a cost-effective approach; (2) these self-proliferating cells are an unlimited source; and (3) they are highly accessible for genetic manipulation (Table 2). However, the lack of standard procedures for the maintenance and differentiation of these cell lines, even though the cell lines are easy to culture, can create variable and sometimes inconsistent experimental results [249,250]. Moreover, due to their cancerous origins, they grow robustly and can lack various relevant attributes/functions typically associated with normal neurons in vivo. For instance, oxidative stress is considered to be an important contributing factor to aging, and ROS perturbation is known to cause senescence and cellular death in cell culture [251,252]. Indeed, H_2_O_2_ treatment of SH-SY5Y, PC-12, or P19-derived neuron-like cells caused a decrease in neuronal survival via caspase-dependent apoptosis [192,193,207]. Nevertheless, no immortalized cell line has been found to have the ability to show a strong expression of various well-established senescence markers, such as p16 and p21, or the clear presence of SA-β-gal activity. However, Kim et al. and Lee et al., when using D-galactose to generate intracellular H_2_O_2_ via galactose oxidase, reported that there was an inhibition of RA-induced SH-SY5Y neuronal differentiation, and that this was accompanied by an increase in SA-β-gal positive cells [187,188]. Overall, the study of cellular senescence using cancer-derived cell lines remains a challenge.

### 3.2. Primary Neuronal Cultures

Primary neurons can be cultured and differentiated into neurons that have a range of neuronal characteristics, including axons, dendrites, dendritic spines, and synapse, and therefore have great potential to overcome many of the difficulties inherent in immortalized cell lines. More importantly, unlike the cell line models described previously, they differentiate without specific inducers [253,254]. They are similar to human neurons in terms of morphology and physiology; primary cultures are thus suitable for investigating neuronal differentiation, aging, and degeneration [255]. The life span of these individual neuronal cells can be prolonged by the use of optimized culture media; they are also amenable to treatments with chemical reagents and may be subjected to electrophysiological investigations [256]. Geng et al. reported that SA-β-gal expression is increased in cultured primary hippocampal neurons at DIV (days in vitro) 20 [197]. Cerebellar granule neurons (CGNs) in culture can survive for 5 weeks, which is then followed by cellular degeneration involving a number of molecular changes, including elevated SA-β-gal activity and an increased intracellular Ca^2+^ level, with the latter suggesting the dysregulation of calcium homeostasis [194]. The sensitivity of CGNs to N-ethyl-N-nitrosourea (ENU) increases in proportion to the age of the culture. Based on the results of alkaline comet assays, there is a reduction in base excision repair (BER) activity as CGNs age in culture. In addition, mitochondrial dysfunction during long-term neuronal culture has been demonstrated by Dong et al. [196]. An age-related reduction in mitochondrial membrane potential (ΔΨm) in neuronal cultures has been found, and so has an increase in ROS generation. These findings indicate that there is an age-related decrease in mitochondrial function, and that the proportion of the senescent cells increases accordingly during the long-term culture of hippocampal neurons. Moreover, aging-associated dysfunctional autophagy has been found to contribute to the senescence transition in neuronal cells, as reported by Moreno-Blas et al. [178]. A higher level of SA-β-gal activity and an accumulation of lipofuscin have been shown to occur during long-term cortical neuronal cultures (DIV40), which have been found to be accompanied by an increased expression of *p21^CIP1/WAF1^* and an increase in γH2AX foci. Nuclear morphology abnormalities and a blockage of autophagy flow, as indicated by the elevated levels of LC3 and p62, have also been detected in aged cortical neurons. Although in vitro primary cultures have provided important clues about the mechanisms behind neuronal senescence, one prominent drawback of these cells is the fact that they are neurons derived from rodents, and thus they can never form a human genome-based model (Table 2).

### 3.3. Human Neurons Directly Converted from Fibroblast (Fib-iNs)

Neuroscientists face a major obstacle when trying to develop drugs to treat brain disorders. Specifically, even when a drug works really well on mice, they still fall short when used to treat humans. One example of this is the development of drugs to treat AD; it has proven to be unusually difficult to do this, with a 99.6% failure rate over the period of 2002–2012 [257]. Currently, the success rate continues at the same low level based on the records of the US Food and Drug Administration (FDA). One of the potential reasons for this is the species gap that exists between rodent models and human studies. The latest studies have shown that the differences between the brains of humans and mice are larger than expected [258,259], which might account for the translation failures when transferring drug treatments for neurological diseases from mice to humans. Therefore, based on the above, many believe that human models are likely to be more accurate at recapturing human physiology and clinical pathology.

The direct conversion of human fibroblasts into induced neurons (Fib-iNs) allows for the generation of human neurons with mature marker expression and functionality [260,261]. The reprogramming of fibroblasts into functional neurons was first achieved by over-expressing a set of transcription factors (TFs), including BRN2, ASCL1, MYT1L, and NEUROD1, all of which are important for the development of neuronal lineages [260,262]. Later, a number of other studies also reported the successful conversion of other cell types into functional neurons by using the same set of TFs [263,264,265,266,267]. The field has since progressed rapidly and has begun to investigate the mechanisms involved in the direct conversion of cells and the identification of the best strategy to use. This has included the use of novel TFs, various microRNAs, and a number of other small molecules for generating a range of neuronal subtypes (comprehensively reviewed in two reviews [268,269]). In contrast to the rejuvenating character of human-induced pluripotent stem cell (hiPSC)-derived neuronal cells (hiPSC-iNs) (see below), Fib-iNs preserve many signatures of aging phenotypes, including transcriptomic, epigenetic, nuclear morphological, mitochondrial, and other aspects of cellular senescence [208,209,210,211], which makes them appropriate for the studies of neuronal aging in vitro. In practical terms, one major drawback of Fib-iNs is their limited cell numbers. Although a variety of cell types have been demonstrated to be converted into iNs, the major source for Fib-iN experiments are adult human skin fibroblasts. Their expandability is limited typically to under twenty passages from an invasive biopsy. The limited starting material makes Fib-iNs inappropriate for a range of later applications, including mechanistic study, drug screening, and transplantation, all of which require relatively large amounts of cells.

### 3.4. Human iPSC-Derived Neurons (hiPSC-iNs)

Due to the extreme sensitivity of the in vitro manipulation of human mature neurons as well as their very limited availability, the study of the human brain at the cellular and molecular level remains a major challenge. In the last decade, research on the hiPSCs have begun to create great hopes for therapeutic application in various diseases and in regenerative medicine [270]. hiPSC-derived and differentiated cells allow researchers to study the impact of a distinct cell type on health and disease, as well as for therapeutic drug screens to be performed on a human genetic background. 

#### 3.4.1. Neuronal Differentiation of hiPSCs

The conventional approach to neuronal differentiation from hiPSCs involves first directing iPSCs differentiation into embryoid bodies and then into neural progenitor cells (NPCs), which is followed by differentiation into functional neurons. The stepwise procedures are principally based on various animal studies of neurodevelopment in which the major events of mammalian neural commitment have been identified. During early embryonic development, neural induction relies on complex interactions between bone morphogenetic protein (BMP) and the transforming growth factor β (TGFβ), fibroblast growth factor (FGF), sonic hedgehog (SHH), Notch, WNT, and RA signaling pathways [271,272,273,274]. These signaling pathways can be modulated by small molecules and/or growth factors in order to direct human embryonic stem cells (ESCs) or iPSCs into neuronal cell fates. For instance, researchers have routinely used FGF2 to induce and then maintain neural progenitor cells starting from the ESC or iPSC stage [275,276,277,278]; this is because FGF signaling have been implicated in the induction and patterning of mesoderm and neural tissues in vertebrate embryos. In 2009, Chambers and Studer’s group described a method by which they differentiated human ESCs or iPSCs into neural cells by dual inhibition of the SMAD signaling pathways [279]. In addition, RA also plays important roles in many aspects of neural development and activity in a developing embryo. In the absence of RA signaling, the posterior hindbrain is not formed and the development of the anterior spinal cord is affected [280]. Similarly, in the dorso–ventral axis of the neural tube, RA works synergistically with other molecules such as the SHH, FGF, BMP, and WNT signaling members, in order to determine the fates of sensory neurons, interneurons, and motor neurons. Thus, RA has been used by many research groups at concentrations of 1–10 μM in medium to bring about hESC neural differentiation [281,282,283,284,285]. The conventional strategy of hESC or hiPSC differentiation into mature neurons usually requires 2–3 months, which mimics human neural development in vivo [286,287]. 

The generation of functional neurons from hiPSCs by direct reprogramming with TFs offers an alternative route for lineage conversion. Previous studies by the Wernig laboratory have shown that a combination of TFs, BRN2, ASCL1, and MYT1L transforms both human ESCs and iPSCs into glutamatergic neurons [260]. Strikingly, the three TFs induced the hiPSCs into neuron-like cells in a short period of time, with the cells exhibiting bipolar morphologies as early as day 3 of the differentiation [260]. The expression of neuronal markers, such as class III β-tubulin (Tuj1) and MAP2, as well as the functional characteristics of action potential generation, were detectable by day 8. Soon after this, the same group reported that hiPSCs can be converted into functional neuronal cells, with nearly 100% yield and purity in less than 2 weeks by the expression of only a single TF such as NEUROG2 or NEUROD1 [288]. The rapid conversion to a neuronal fate of cells has demonstrated that there is a faster path to TF-based neural induction compared to conventional differentiation using growth factors or signaling molecules. Subsequently, our group and others have reported distinct sets of TFs that could promote ESC or iPSC differentiation towards numerous cell types in the brain, including glutamatergic, dopaminergic, GABAergic, serotonergic, and motoneurons [288,289,290,291,292], as well as astrocytes, oligodendrocytes, and microglia [293,294,295]. This reprogramming strategy permits the cells to skip the progenitor stage and accelerates the time course of neuronal differentiation and maturation. This presents a unique opportunity to understand how the aging process impacts these cells and how various neurodegenerative disorders occur.

#### 3.4.2. Approaches for Carrying out an In Vitro Aging-like Process Using hiPSC-Derived Neurons

In contrast to Fib-iNs, hiPSCs, once they are successfully converted from somatic cells, can theoretically expand infinitely. These properties make hiPSC differentiation suitable for the generation of large numbers of neurons and their subsequent application in mechanistic studies, drug screening, and transplantation, all of which require relatively large amounts of material. However, the rejuvenation effect of iPSC reprogramming is a major drawback when attempting to model age-related diseases [211,268,296]. After iPSC reprogramming has occurred, many signs of cellular aging such as nuclear envelope integrity and mitochondrial function are improved, and the aged epigenome is reset to zero [297,298,299,300]. The telomeres of iPSCs are also longer than in the parent differentiated cells, and are comparable in length to the telomeres of control ESCs [301]. 

**Figure 2:**
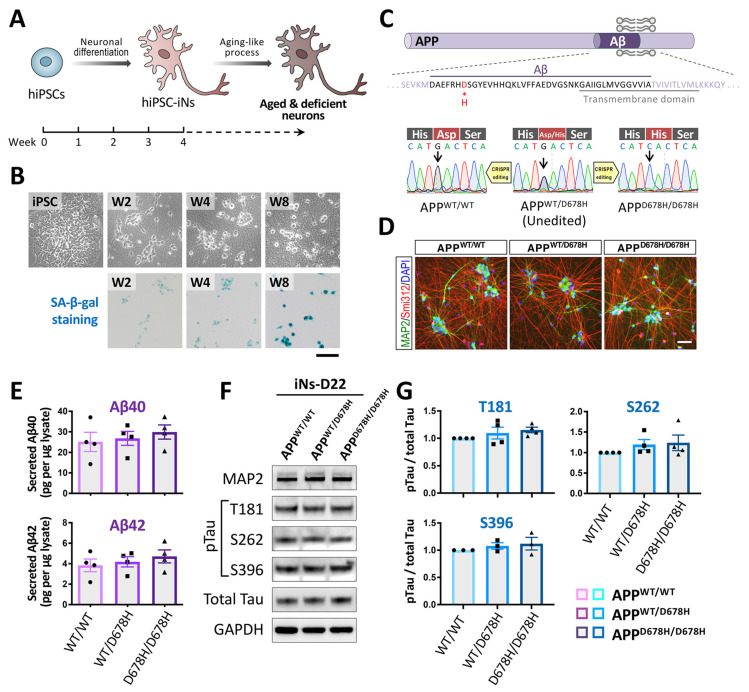
The early mature hiPSC-iNs derived from an AD patient show no significant changes in neuronal morphology and AD pathological characteristics. (**A**) The hiPSCs are induced to differentiate into neuronal cells by the Ngn2 overexpression method. Senescence-like features can be elicited by various strategies in mature hiPSC-iNs. (**B**) SA-β-gal activity is increased in the hiPSC-iNs after a prolonged culture of 8 weeks. Scale bar, 50 μm. (**C**) The diagram presents human APP protein and the D678H (the 678th amino acid using the APP770 numbering) mutation within the Aβ region of AD-iPSC (upper panel). Direct sequencing of the APP exon 16 PCR products derived from the patient revealed a GAC-to-CAC nucleotide substitution in the Aβ region of the patient’s APP gene. The CRISPR-edited isogenic hiPSC clones were confirmed by Sanger sequencing (lower panel). (**D**) Representative images of isogenic iPSC-derived glutamatergic neurons immunolabeled for the dendritic and axonal markers, MAP2 and Smi312, respectively, at day 22. Scale bar, 50 μm. (**E**) The amount of Aβ40 and Aβ42 secreted from the isogenic iPSC-derived neurons (n = 4). (**F**) Western blot analysis was used to monitor the expression of phosphorylated tau at T181, S262, S396, and total tau in the isogenic iPSC-derived neurons. GAPDH (glyceraldehyde-3-phosphate dehydrogenase) was used to confirm similar protein loadings across the samples. (**G**) Quantitative results of (**F**). The intensity of the pTau signals was normalized against total Tau.

In fact, iPSC-based disease models derived from patients with neurodegenerative disorders typically exhibited no disease-associated phenotypes under normal culture conditions [213,214,302]. Our group also demonstrated that hiPSC-iNs derived from an AD patient with the APP_D678H mutation show no significant changes in neuronal morphology, gene expression levels, and AD pathological characteristics, such as Aβ accumulation and Tau protein hyperphosphorylation, during differentiation and early maturation, even compared to its isogenic controls (Figure 2C–G). These observations suggest that patient iPSCs can successfully differentiate into mature iNs under normal conditions, and may only mimic the phenotypic features at the pre-clinical stage of a disease or perhaps at an early stage of a disease. As aging is the primary risk factor for AD, the patient iPSC-iNs might recapitulate AD-like pathologies only when they are switched to an aged status. 

In the last few years, researchers have used various strategies to express a relevant phenotype for neurodegenerative disorders in iPSC models. For example, Lorenz Studer and colleagues demonstrated that aging characteristics can be reintroduced into iPSC-derived neurons by overexpressing progerin, a mutant form of the lamin A (LMNA) protein that is responsible for the premature aging disorder Hutchinson–Gilford Progeria [213]. Using this type of approach, dopaminergic neurons derived from Parkinson’s patient iPSCs have been shown to develop increased disease-associated phenotypes, including the folding and blebbing of the nuclear membrane, the formation of DNA double-strand breaks, mitochondrial ROS (mtROS), and the breakdown of established neurites. The same group also developed an alternative and potentially more physiological approach to induce aging; they did this by pharmacologically inhibiting telomerase activity [214]. These hPSC-derived neurons with shorter telomeres present with aged-like features such as increased DNA damage, mtROS, and reduced dendrite numbers. In addition, the induction of DNA damage using chemical stressors has been shown to result in dysfunctional DNA damage repair and other age-related features, including augmented SA-β-gal staining, decreased proliferation, and reduced differentiation capacity, as well as an elevated level of ROS [212]. Thus, even though age-inducing strategies have successfully recapitulated certain types of pathological aging in iPSC derivatives, which represent a significant step forward in modeling age in vitro with iPSCs, it remains unclear whether these approaches imitate normal human aging. More recently, our group found that SA-β-gal activity increased as a function of time upon the prolonged culture of hiPSC-iNs for 8 weeks (Figure 2B). It will be interesting to determine to what extent these different strategies may be combined to provide a more complete picture of actual cellular senescence using human neurons in vitro.

## 4. Application of Genome Editing Technology to hiPSC-Derived Neurons in Order to Study Cellular Senescence and Carry out Drug Discovery

Genetic manipulation, the process of inducing changes in gene expression level or introducing particular gene variants, has proven to be an indispensable tool in recent biomedical research. Previous conventional methods of genomic sequence replacement in mammalian cells using exogenous donor DNA via homologous recombination (HR), and the correct insertion of exogenous DNA do allow the targeted insertion of virtually any DNA sequence in a genome, but the reported efficiencies are low (<1%). To address this need, the advent of technologies using DNA-binding zinc-finger nuclease (ZFN) and transcription activator-like effector nucleases (TALENs) has greatly improved the efficiency of the genome editing of mammalian cells [303,304]. Furthermore, clustered regularly interspaced short palindromic repeats (CRISPR) with the CRISPR-associated protein (Cas9) system have been demonstrated to have revolutionary potential when engineering the genome of cultured cells and animal model systems; they are capable of carrying out the editing at a much high efficiency [305,306]. The latter approach has become a powerful tool for biological research and has opened up a new therapeutic avenue for the treatment of neurological diseases. Briefly, the CRISPR/Cas9 system contains two components: a single-guide RNA (sgRNA) and an endonuclease (Cas9). The sgRNA/Cas9 complex is able to bind to a specific locus on the genome, and it then induces a double-strand DNA break (DSB), which is subsequently repaired by two of the cells’ major repairing mechanisms, namely non-homologous end-joining (NHEJ) and homology-directed repair (HDR) [307]. During the process of DNA repair, the targeted DNA sequence can be modified by the introduction of a small insertion and/or deletion (indel) mutation using NHEJ, or by the insertion of a functional DNA fragment using HDR. The CRISPR/Cas9 system has proven to be highly efficient at gene disruption, by knocking in a gene or DNA fragment, or correcting genetic defects in various human cells [308]. For genetic functional screening, a new approach has been created from the CRISPR/Cas9 system; this approach uses a catalytically inactive Cas9 (dCas9) that is fused to either a transcription repressor (KRAB) [309,310,311] or a transcription activator (VP64) [312,313,314]. The result is either gene repression (CRISPR interference, CRISPRi) or gene activation (CRISPR activation, CRISPRa) in a mammalian cell system, including hiPSCs.

### 4.1. The CRISPR/Cas9-Mediated Genome Editing and Gene Expression in hiPSCs

Accompanied by the development of hiPSC-derived neuronal models, gene editing technologies have significantly improved our understanding of age-associated neuronal senescence, as well as of neurodegenerative disorders. The use of hiPSCs from patients to generate disease models has provided an invaluable tool for studying the early pathogenic mechanisms acting in neurological disorders that have monogenic variant/mutation. In order to compare phenotypic effects robustly in vitro, an isogenic line, in which the known gene mutation is corrected by the CRISPR/Cas9 method, is necessary in order to ensure that the observed differences are attributable to a specific genetic defect. For example, we generated hiPSC lines from AD patients that carry the APP mutation as well as gene-corrected isogenic clones, and then used these to assess the therapeutic potential of a Chinese herbal medicine [302]. Moreover, CRISPR/Cas9 was also utilized to create mutations in iPSC lines from healthy control subjects. This was particularly helpful when exploring rare diseases where there is reduced accessibility to patient-derived samples that allow for the generation of patient-derived iPSCs, or when there is difficulty in obtaining patient samples with a specific genotype. The genome editing of isogenic lines also allowed the study of many variants with the same genetic background at once, which can be more practical than gathering a large number of patient and control lines. 

More recently, the integration of CRISPR-based functional genomics and stem-cell technology has enabled the scalable interrogation of gene function in human neurons. The CRISPR-based platform overcomes the greatest constraint in this area, namely the off-target effects associated with the conventional RNA interference (RNAi) approach when carrying out genome-wide functional screening. For instance, Tian et al. reported the first genome-wide CRISPRi and CRISPRa screens targeting hiPSC-derived neurons and uncovered several pathways that control neuronal survival and the response of cells to oxidative stress [315,316]. Liu et al. developed a CRISPRa approach to systematically identify the regulators of neuronal-fate specification [317]. To investigate the genetic and epigenetic bases of cellular aging, Wang and colleague carried out a CRISPR/Cas9-based screen that identified KAT7 (a histone acetyltransferase gene) as a driver of cellular senescence in human mesenchymal precursor cells [318]. The prioritization of genes identified from functional genomic screens seems to be a good approach in the development of anti-aging interventions and therapies. 

### 4.2. The Inducible CRISPR/Cas9 Systems in hiPSC-iNs

**Figure 3:**
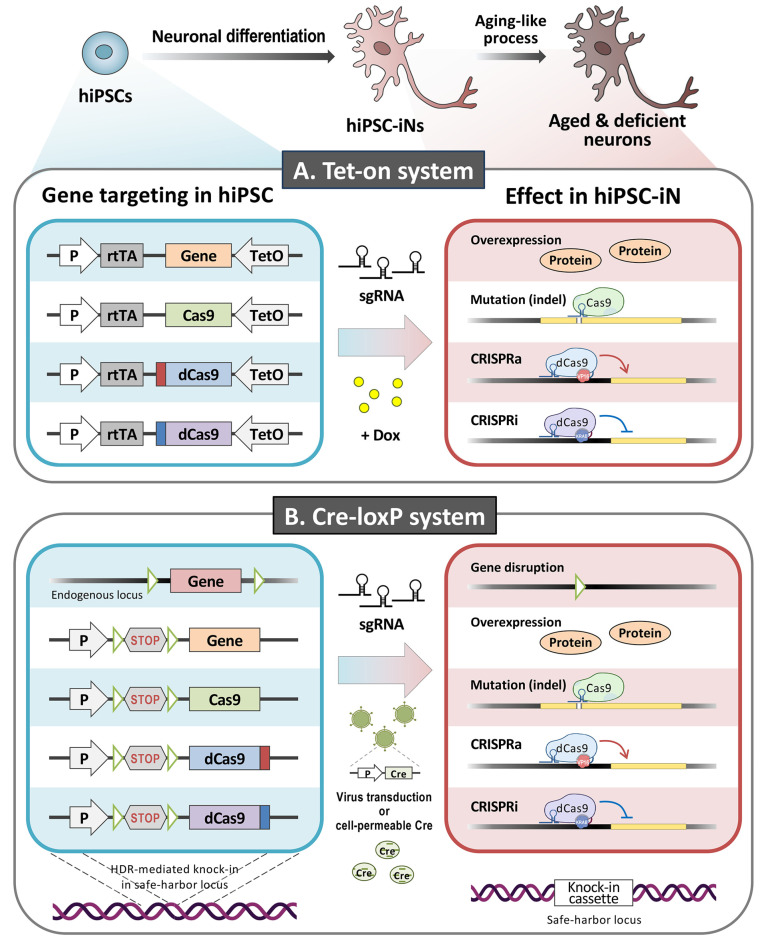
Schematic of the inducible system in hiPSC-iNs. The inducible functional cassette is knocked into a genomic safe harbor (e.g., AAVS1) in the hiPSCs via CRISPR/Cas9-mediated homology-directed repair (HDR). The temporal control of gene expression or inactivation is achieved by the addition of doxycycline (Dox) (**A**) or Cre protein (**B**), respectively, either to the hiPSCs or the hiPSC-iNs. The figure was created with Biorender.com accessed on 19 October 2021.

Protein overexpression and gene knockdown or knockout are key technologies when studying molecular mechanisms in neuroscience research. However, conventional genetic manipulation methods are inadequate when studying senescence processes in mature neurons as they may cause early lethality at the hiPSC or neural progenitor stage. It has been suggested that fine-tuning the expression of some lethality-causing genes or essential genes in differentiated (and aged) neurons is necessary in order to be able to analyze their functions. Nevertheless, post-mitotic neurons are generally difficult to culture and are particularly resistant to the delivery and expression of recombinant genes, thereby often limiting experimental approaches. To address this issue, an inducible approach that will allow the genetic manipulation of mature neurons would be highly desirable in order to facilitate research on the molecular basis of neuronal function, especially the aging process (Figure 3). The available tools, namely inducible systems such as Tet-on and Cre-loxP, have been used extensively for gene regulation using in in vitro and in vivo models [319]. Briefly, the inducible functional cassette is knocked into a genomic safe harbor (e.g., AAVS1) in hiPSCs via CRISPR/Cas9-mediated HDR. After neuronal differentiation, temporal control of gene expression or gene inactivation in the mature hiPSC-iNs is achieved by the addition of either doxycycline (Dox) or Cre protein, respectively. Recently, Ding and colleague reported that they were able to generate a human gene-editable iPSC line that could be used to efficiently introduce specific mutations through the addition of designed sgRNAs, and these modified cells could then be induced to differentiate into disease-modeling cells [320]. The inducible system using CRISPR technology, combined with the hiPSC-iN approach, is becoming a valuable tool for the study of molecular mechanisms during neuronal senescence, as well as being useful for drug discovery. 

## 5. Conclusions and Perspectives

Neurons are among the longest living cells in the human body. Most of them are generated during embryonic development, with only a few new neurons being produced after birth [321]. Neurons from primary human sources are also exceptionally difficult to obtain and maintain, which makes their direct study highly challenging. Thus, hiPSC-iNs generated from human donors present a unique opportunity to begin to understand how the aging process impacts these cells and then gives rise to various neurodegenerative diseases. This review summarizes the key features of cellular aging that appear to drive neurodegeneration, compares the currently available in vitro platforms, and highlights various hiPSC-iN approaches that are being used for next-generation human cellular modeling. Although hiPSCs are largely rejuvenated and have erased most aging signatures, we have demonstrated that the senescence-like features can be elicited in mature hiPSC-iNs upon prolonged culture (Figure 2B). 

**Figure 4:**
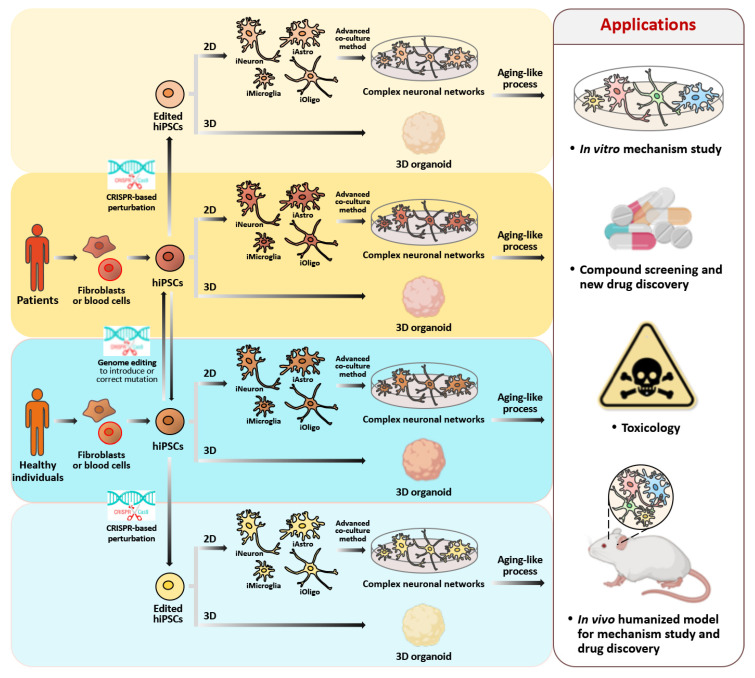
Schematic representation of the workflow of hiPSC generation, genome editing, and neuronal differentiation from an individual’s somatic cells, and their major applications in the area of human healthy aging. A healthy person’s or patient’s somatic cells are reprogrammed into a pluripotent stage. To generate isogenic control iPSCs for the follow-up analyses, the mutation in the patient’s iPSCs can be corrected using CRISPR-mediated editing. Similarly, a specific disease-causing mutation can be introduced into the genome in the control hiPSCs in order to allow for disease modeling. These hiPSC lines are then differentiated into disease-associated cell types in a 2D environment; these cell types can include neurons, astrocytes, oligodendrocytes, or microglia. Alternatively, they can be differentiated into brain organoids in a 3D environment. Following an aging-like process, the hiPSC-derived brain cells display a number of senescence phenotypes that can be assayed in vitro. Furthermore, the hiPSC derivatives can be used for drug screening, for the discovery of new drugs, for toxicity testing, and as models for the study of the mechanisms involved in human neuronal aging in vitro and in vivo. The figure was created with Biorender.com accessed on 19 October 2021.

In fact, the brain is composed of a variety of cell types, including neurons, astrocytes, oligodendrocytes, microglia, and endothelial cells, all of which work in concert to perform activities throughout the whole human lifetime. The effects of aging vary depending on the brain region of interest and the different cell types present, and cells may sometimes even move in the opposite direction to normal aging [322]. Thus, co-cultures of neurons and other types of cells are extremely useful tools in vitro when evaluating cell-to-cell interactions that rely on direct contact or the release of soluble factors. This makes them a suitable method for the study of the contribution of various cell types to neuronal senescence. Human cerebral organoids have recently emerged as a state-of-the-art technology for modeling human brain development in three-dimensions (3D). This may eventually represent an alternative strategy for the better recapitulation of human brain aging in vitro [323]. In summary, there is currently great interest in determining the extent to which various available strategies can be combined in order to provide a more complete picture of actual cellular senescence in human neurons (Figure 4).

In this review, we propose that the iPSC-to-iN paradigm should be able to provide a new platform for the study of the mechanisms associated with brain development, brain maturation, and brain aging at the cellular level. In addition, because hiPSCs are reprogrammed and rejuvenated from human adult cells by a complete erasing of age-associated and senescence-associated features such as DNA methylation patterns, they also represent a proof of concept that many aspects of cellular aging are reversible. Consequently, such a platform could have important implications for the identification of drug targets that can slow down or potentially reverse aging phenotypes.

## Figures and Tables

**Table 1:** The characteristics of the in vitro cell models that can be used to study neuronal senescence.

	Cell Line [Ref]	Source	Method of Generating Neurons	Characterization of Neurons	Method of Inducing Cellular Aging (Reagent/Time)	Phenotypes of Cellular Aging
**Immortalized cell lines**	**SH-SY5Y** (ATCC CRL-2266) [187,188,189]	Human neuroblastoma	– RA – RA+BDNF – RA+TPAF – RA+dbcAMP – NGF	Marker expression: – Pan-neuronal: Tuj1, NeuN, NSE, neurofilaments – Dendritic: MAP2 – Axonal: Tau, SMI312 – Synaptic: synapsin I, synaptophysin, PSD-95 Functional characterization: – Calcium activity – Action potential	H_2_O_2_/24 h D-galactose/2~3 days	H_2_O_2_ – Cell viability↓ – LDH ↑ – Caspase 3 activity↑ – Mitochondrial membrane potential (ΔΨm) ↓ – ROS level ↑ – Autophagy ↑ D-galactose – Cell viability ↓ – Growth inhibition – Growth inhibition – Autophagy ↑ Involvement of necroptotic pathway
	**Neuro-2a** (ATCC CCL-131) [190,191]	Mouse neuroblastoma	– RA – dbcAMP – TPA			
	**PC-12** (ATCC CRL-1721) [192]	Rat adrenal gland	– NGF			
	**P19** (ATCC CRL-1825) [193]	Mouse embryonic carcinoma	– RA			
**Primary neuronal cultures**	**Primary** **neurons** [178,189,194,195,196,197,198,199,200,201,202,203,204,205,206,207]	Rodent (mouse or rat) hippocampus or cortex	– Animal dissection and appropriate culture conditions		D-galactose/~12 DIV Long term culture/ 6~35 DIV	Long-term culture – SA-β-gal activity ↑ – Growth inhibition – Intracellular Ca^2+^ ↑ – BER ↓ – TopoIIβ expression ↑ – ΔΨm ↓ – ROS ↑ – p21^CIP1/WAF1^ ↑ – γH2AX foci ↑ – Nuclear membrane abnormalities – Autophagosome accumulation
**Human** **ibroblast (hFib)-** **converted** **neurons**	**hFib-** **converted neurons** [208,209,210,211]	Human fibroblasts from donor individuals	– Using transcription factors – Using microRNAs and small molecules		Preserve age-associated features	Epigenetic modification
**Human** **induced** **pluripotent stem cell (hiPSC)** **-derived** **neurons**	**hiPSC-** **derived** **neurons** [212,213,214]	Human iPSCs derived from donor individuals	– Embryoid body formation – Using microRNAs, small molecules and growth factors – Using transcription factors		– Expression of progerin – Telomerase inhibitor at iPSC stage – Application of stressors	– Nuclear morphology abnormalities – Loss of the nuclear lamina-associated proteins – Formation of DNA double strand breaks (γH2AX) – Mitochondrial dysfunction – Neurite degeneration

Note. RA, retinoic acid; BDNF, brain-derived neurotrophic factor; TPA, tetradecanoylphorbol-13-acetate; dbcAMP, dibutyl cyclic AMP; NGF, nerve growth factor; LDH, lactate dehydrogenase; ROS, reactive oxygen species; BER, base excision repair.

**Table 2:** The advantages and disadvantages of the various in vitro cell models used to study neuronal senescence.

	Benefits	Drawbacks
**Immortalized cell lines**	– Easier to work with and cost-effective – Proliferating cells – Accessible for genetic manipulation	– Need to be induced into neuron-like cells – Immortalized with dysregulated cell cycles and cancer phenotypes
**Primary neuronal cultures**	– Genetically more stable than neuronal cell lines – Maintain many crucial markers and functions as observed in vivo	– Non-human
**Human fibroblast (hFib)-converted neurons**	– Epigenetic aging is maintained – Able to convert into various neuronal subtypes – Time efficiency (skip hiPSC generation)	– The starting cell source is limited – Need to be induced into neuron-like cells – Costly
**Human** **induced** **pluripotent stem cell (hiPSC)** **-derived** **neurons**	– Theoretically expand infinitely – Able to differentiate into various neuronal subtypes – 3D organoids – Accessible for genetic manipulation	– Largely rejuvenated – Need to be induced into neuron-like cells – Costly

## Data Availability

Not applicable.
